# Structural insights of a hormone sensitive lipase homologue Est22

**DOI:** 10.1038/srep28550

**Published:** 2016-06-22

**Authors:** Jing Huang, Ying-Yi Huo, Rui Ji, Siyun Kuang, Chaoneng Ji, Xue-Wei Xu, Jixi Li

**Affiliations:** 1 State Key Laboratory of Genetic Engineering, Collaborative Innovation Center of Genetics and Development, Shanghai Engineering Research Center of Industrial Microorganisms, School of Life Sciences, Fudan University, Shanghai, 200438, China; 2 Key Laboratory of Marine Ecosystem and Biogeochemistry, Second Institute of Oceanography, State Oceanic Administration, Hangzhou, 310012, China

## Abstract

Hormone sensitive lipase (HSL) catalyzes the hydrolysis of triacylglycerols into fatty acids and glycerol, thus playing key roles in energy homeostasis. However, the application of HSL serving as a pharmaceutical target and an industrial biocatalyst is largely hampered due to the lack of high-resolution structural information. Here we report biochemical properties and crystal structures of a novel HSL homologue esterase Est22 from a deep-sea metagenomic library. Est22 prefers short acyl chain esters and has a very high activity with substrate *p*-nitrophenyl butyrate. The crystal structures of wild type and mutated Est22 with its product *p*-nitrophenol are solved with resolutions ranging from 1.4 Å to 2.43 Å. The Est22 exhibits a α/β-hydrolase fold consisting with a catalytic domain and a substrate-recognizing cap domain. Residues Ser188, Asp287, and His317 comprise the catalytic triad in the catalytic domain. The *p*-nitrophenol molecule occupies the substrate binding pocket and forms hydrogen bonds with adjacent residues Gly108, Gly109, and Gly189. Est22 exhibits a dimeric form in solution, whereas mutants D287A and H317A change to polymeric form, which totally abolished its enzymatic activities. Our study provides insights into the catalytic mechanism of HSL family esterase and facilitates the understanding for further industrial and biotechnological applications of esterases.

Lipolytic enzymes, including esterases (EC 3.1.1.1) and lipases (EC 3.1.1.3), are carboxylicester hydrolases (EC 3.1.1), which catalyze the hydrolytic cleavage of an ester bond between a carboxylic acid and an alcohol group in water^1^,^2^. With high substrate specificity and chiral selectivity, lipolytic enzymes are extensively used in the synthesis and product of biopolymers, biodiesel, chiral pharmaceutical compounds, and agrochemicals^3^,^4^. Hormone-sensitive lipase (HSL), one member of lipolytic enzymes, mediates the hydrolysis of triacylglycerols to provide free fatty acids and glycerol^5^. HSL plays essential roles during nutrient deprivation and energy supply in the human body, influencing the pathogenesis of type II diabetes, obesity, and cardiovascular disease^6^,^7^,^8^. As HSL has broad substrate specificity, it also serves as a potential environment and industrial biocatalyst^9^.

The human HSL is a multiple domain protein, composed of a tolA domain in the N-terminal region, an HSL_N domain and alpha/beta hydrolase domain in the central region, and a C-terminal end. The HSL_N domain interacts with other proteins, whereas the alpha/beta hydrolase domain catalyzes the hydrolysis of lipid substrates. The hydrolase domain of human HSL shares homologous sequence identity and a conserved motif with the IV family of lipolytic enzymes^9^. Thus far, there are no high-resolution structures of human HSL members available due to the failure of protein crystallization. However, several atomic structures of the lypolytic IV members have been reported, including EstE1, EstE7, and Est25 from metagenomic libraries^10^,^11^,^12^, Sto-Est from *Sulfolobus tokodaii*^13^, and Brefeldin A Esterase (BFAE) from *Bacillus subtilis*^14^.

Recently, we identified a new bacterial esterase homologue Est22 (Genbank ID: JF766290) of the HSL family from a deep-sea sediment 5,000 meters under water in the Pacific ocean^15^. In this study, we analyzed the biochemical properties and crystal structures of Est22 with its product *p*-nitrophenol in order to obtain a broader understanding of the HSL-family enzymes and to investigate the potential uses of Est22 as a biocatalyst. Est22 has a very high enzymatic activity of 2,065 U/mg with the substrate *p*-nitrophenyl butyrate, and exhibits a characteristic α/β-fold structure. Investigation into the different states of the wild type and mutated Est22 structures greatly improve our understanding of the molecular mechanism of the HSL family.

## Results and Discussion

### Biochemical properties of the esterase Est22

**Figure 1:**
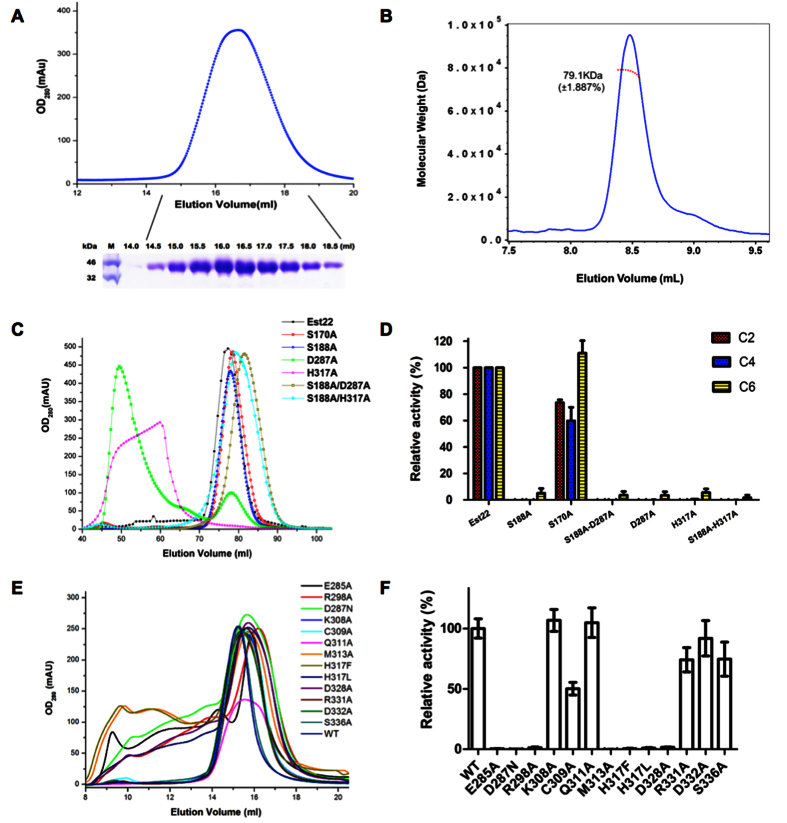
Gel filtration profiles and enzymatic activities of Est22 and mutants. (**A**) (top) Gel filtration profile of Est22 on a Superdex 200 10/300 column. The Est22 was eluted at peak 16.6 mL. (bottom) SDS-PAGE of Est22 protein eluted from gel filtration. The left lane was the molecular weight marker (labeled in kDa). (**B**) Multi-angle light scattering (MALS) analysis of Est22 on the wtc030s5 column (Wyatt) with the elution buffer (20 mM Tris-HCl, 100 mM NaCl, pH 7.4). The molecular weight of Est22 was 79.1 kD, which showed that Est22 is a dimer in solution. (**C**) Gel filtration profile of Est22 and mutants on a Superdex 200 16/600 column. Wild type Est22 and other mutants S170A, S188A, S188A/D287A, and S188A/H317A formed dimers in solution, whereas mutants D287A and H317A changed from dimeric to polymeric forms evidenced as they were eluted in void volume on gel filtration profiles. (**D**) Esterase activity was examined using different substrates, including *p*-nitrophenyl acetate (C2), *p*-nitrophenyl butyrate (C4) and *p*-nitrophenyl hexanoate (C6) at pH 7.5 and 40 °C. Compared with wild type Est22, S170A mutant decreased 10–30% activity with different substrates, whereas other mutants almost abolished the enzyme activities with substrates. (**E**) Gel filtration profiles of WT Est22 and mutants. The amino acid residues that involved in dimeric surface were mutated to alanine. Mutants K308A, C309A, Q311A, M313A, R331A, D332A, and S336A changed slightly, whereas mutants E285A, R298A, M313A, D328A, D287N, H317F and H317L showed heterogeneity on gel filtration, distributing from dimeric to polymeric states. (**F**) The enzymatic activities of WT Est22 and mutants were examined with C4 substrate.

In order to investigate the biochemical properties of Est22, we expressed the recombinant Est22 protein with a 6xHis tag in *Escherichia Coli*. The Est22 was purified into 95% homogeneity after Ni-NTA affinity chromatography and gel-filtration chromatography. The molecular weight of Est22 is 79.1 kDa on gel filtration profile evidenced by the multi-angle light scattering (MALS) method. As the theoretical MW for 6xHis fusion Est22 is 38.9 kDa, this shows that Est22 is a dimer in solution (Fig. 1a,b).

**Figure 2:**
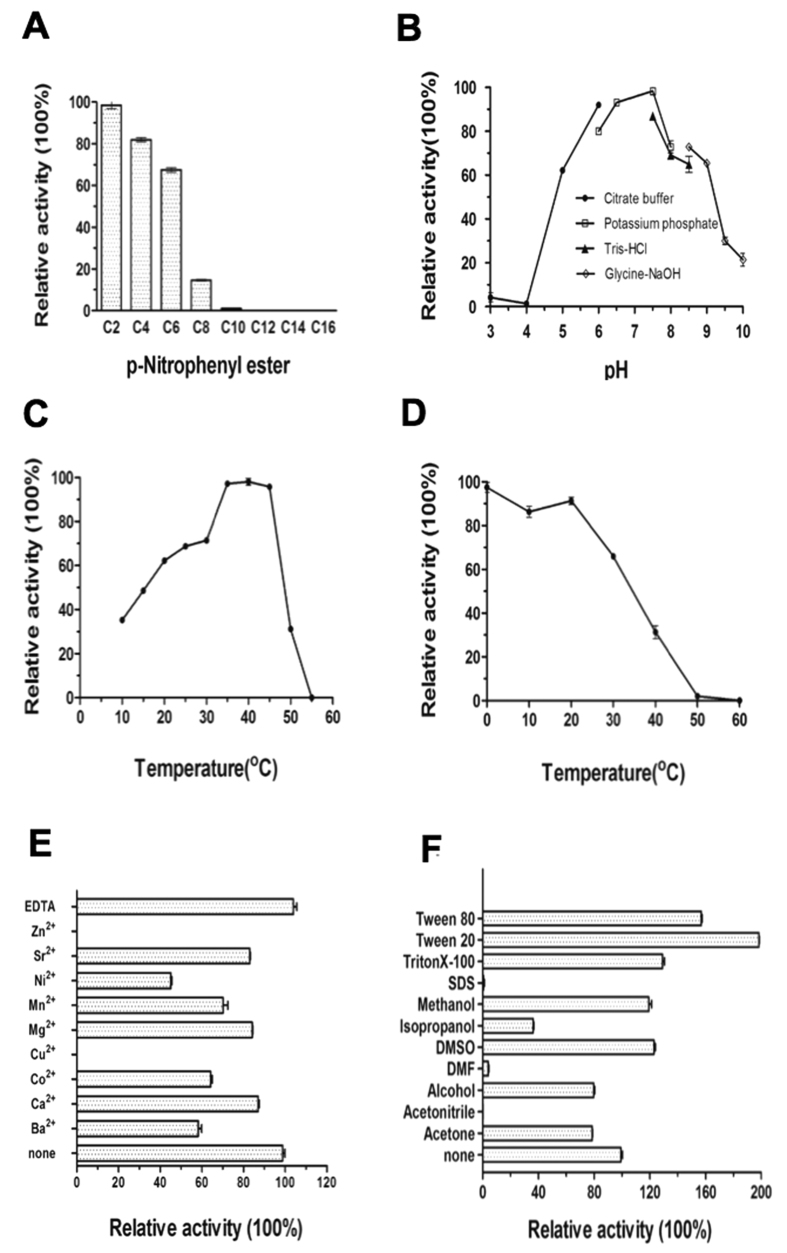
Biochemical properties of Est22. (**A**) Substrate specificity of Est22. The esterase activity of Est22 on *p-*NP esters with various chain lengths was assayed at 40 °C, pH 7.5. The highest level of activity with the *p*-nitrophenyl acetate (C2) substrate is shown as 100%. (**B**) Effect of pH on the activity of Est22. The activity was measured with substrate C2 at 40 °C in different buffers: 100 mM citrate buffer (pH 3.0–6.0) (*filled circle*), 100 mM potassium phosphate buffer (pH 6.5–8.0) (*empty square*), 100 mM Tris-HCl buffer (pH 7.5–8.5) (*filled triangle*), and 50 mM CHES buffer (pH 8.5–10.0) (*empty diamond*). (**C**) Effect of temperature on the activity of Est22. Enzyme activity was measured under various temperatures at pH 7.5 with substrate C2. The value obtained at 40 °C is shown as 100%. (**D**) Thermostability of the recombinant Est22. (**E**) Effects of metal ions on the activity of Est22. An enzymatic assay was performed at 20 °C in100 mM Tris-HCl buffer (pH 7.5) with substrate C2. Metal ions (Zn^2+^, Sr^2+^, Ni^2+^, Mn^2+^, Cu^2+^, Co^2+^, Ca^2+^, Mg^2+^, and Ba^2+^) and EDTA were added at the final concentration of 10 mM. (**F**) Effects of detergents and organic solvents on the activity of Est22. Organic solvents were added at the concentration of 15%. Detergents (Tween 20, Tween 80, Triton X-100, and SDS) were added at the concentration of 1%. The value obtained with no additives in the reaction mixture is shown as 100%. Enzyme activity was measured under standard condition.

The enzymatic activity of Est22 was analyzed in 100 mM Tris-HCl buffer pH 7.5 at 40 °C by using *p*-nitrophenyl acetate as a substrate. We found that the reaction rate of Est22 is 2,065 U/mg, while the Km, Vmax, and kcat values are 0.4899 mM, 44.02 μM/min, and 3160 s^−1^, respectively. All of these values are significantly higher than previous reported esterases^10^,^11^,^12^. The substrate specificity of Est22 was examined using various *p*-nitrophenyl (*p-*Np) esters with acyl chain lengths from C2 to C16 in standard conditions. We found that Est22 hydrolyzed *p*-Np esters up to C8, while the enzymatic activity decreased significantly with increasing chain length of the *p*-Np esters. Est22 had a maximal enzymatic activity at pH 7.5 and at 40 °C, whereas the enzymatic activity of Est22 slightly decreased to 95% at 35 °C. Thermostability analysis showed that Est22 activity decreased with increasing temperature, and was inert beyond 50 °C (Fig. 2a–d).

As investigation into the tolerance of Est22 to different metal ions, detergents, and organic solvents is critical for industrial applications, therefore, nine divalent cations were utilized during the enzymatic activity test. Zn^2+^ and Cu^2+^ abolished the enzymatic activity of Est22. Ni^2+^, Co^2+^, and Ba^2+^ had decreased activity upwards of 70%, whereas the other four cations (Sr^2+^, Mn^2+^, Mg^2+^, and Ca^2+^) had little effect. The chelating agent EDTA had no obvious inhibition on enzymatic activity, which indicated this esterase was not a metal enzyme (Fig. 2e). These results suggest that Est22 has the potential to be applied in the industrial environments containing metal ions such as Sr^2+^, Mn^2+^, Mg^2+^, and Ca^2+^. Next, we investigated the influence of organic solvents and detergents to the enzyme activity of Est22 (Fig. 2f). We found that the activity of Est22 was completely abolished in 1% SDS and 15% acetonitrile, while there was a decrease in enzymatic activity (from 20% to 90%) in 15% acetone, alcohol, dimethylformamide (DMF) and isopropanol. However, 15% dimethyl sulfoxide (DMSO), methanol, 1% TritonX-100, Tween 20, or Tween 80 can increase its activity (Fig. 2f).

### The monomer structure of Est22

**Figure 3:**
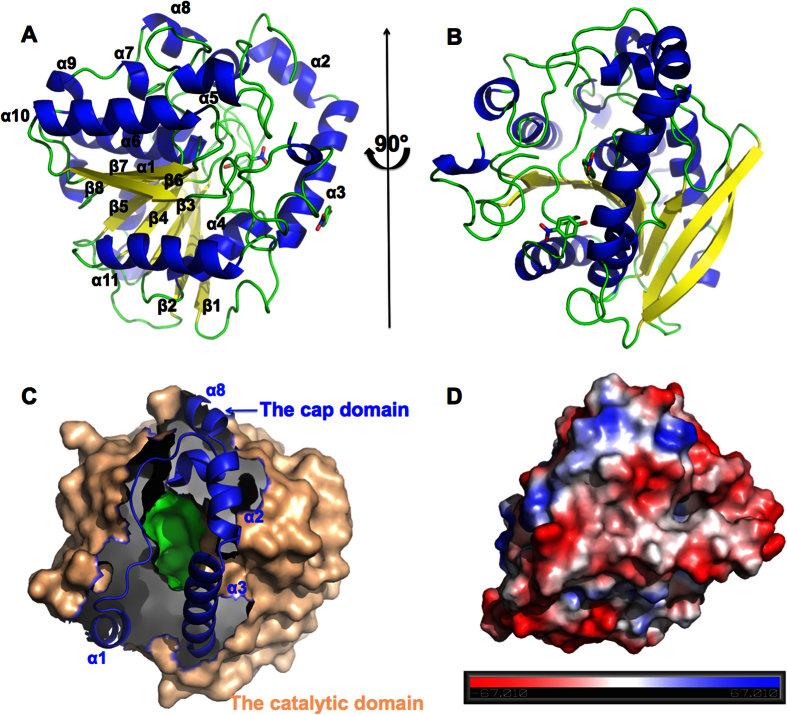
Structure of monomeric Est22 bound with the product *p*-Np. (**A**,**B**) Ribbon representation of Est22 bound with the product *p*-Np, showing 11 α-helices and 8 β-sheets. The two *p*-Np molecules are shown as a ball-and-stick model. (**C**) The cap domain is shown as cartoon in dark blue, while the catalytic domain surface is colored in wheat. The substrate-binding pocket is shown in green. (**D**) The electronic potential surface of Est22 monomer. Red: negative potential; blue: positive potential.

The crystal structures of wild type and mutated Est22 and with its associated product *p*-Np, were solved with resolutions ranging from 1.4 Å to 2.43 Å. Given that the overall architecture is very similar among these structures, we will discuss below the product *p*-Np bound Est22 structure (Fig. 3a,b). The crystal structure of Est22 with its product *p*-Np was refined to 1.4 Å resolution with a satisfied R factor and R_free_ factor values of 14.27% and 16.68%, respectively (PDB ID: 5HC0). The crystallographic statistics for data collection and structure refinement are summarized in Table 1. The Est22 exhibits a classical α/β-fold hydrolase structure, which is composed of 11 α-helices and 8 β-sheets (Fig. 3a). There are two *p*-Np molecules in this structure. One is outside of the cap domain, which could be due to non-specific binding. The other is in the substrate-binding pocket, but it does not occupy the active site (Fig. 3a). The calculated surface electrostatic potential of Est22 is negatively charged (Fig. 3d).

The Est22 monomer structure can be divided into two domains: a catalytic domain with amino acid residues of Ala58-His243 and Met258-Gly344 and a cap domain with amino acid of α1 (Pro13-Phe19), α2 (Arg33-Ala40), α3 (Glu43-Asp57) and α8 (Gln246-Glu257) (Fig. 3c). The catalytic domain has a canonical α/β-hydrolase fold structure: a central β-sheet having eight almost parallel strands together with surrounded α-helices. As has been shown in other structures, this architecture provides a stable scaffold for active sites^10^. The central β-sheet displays a left-handed super helical twist, where β1 and β8 strands cross between each other at ~120° (Fig. 3a). There are six α-helices, namely α4 (Gly118-Ala128), α5 (Ala156-Leu175), α6 (Gly189-Leu209), α7 (Pro230-Glu234), α9 (Glu270-Vla273), and α10 (Arg290-Arg302), packing on both sides of the β-sheets. The cap domain is located on the top of the substrate-binding pocket of Est22, which has the ability to recognize substrates, and can control the substrate entry by maintaining the structural integrity around the binding pocket (Fig. 3c)^10^. The amino acid sequence of the cap domain is poorly conserved, but is structurally similar to other esterases/lipases of HSL family esterases.

### The substrate binding pocket and active sites of Est22

**Figure 4:**
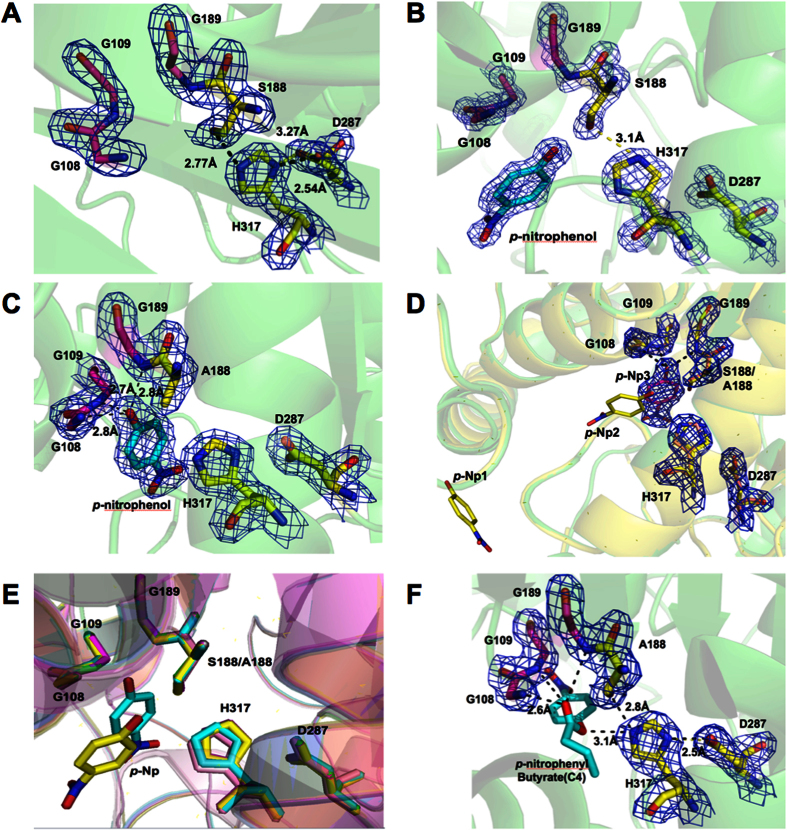
Visualization of substrate-binding pocket and active site of Est22. (**A**) The residues of the catalytic triad and the oxyanion hole in wild type Est22 are shown as stick models. Residue His317 forms hydrogen bonds with Ser188 and Asp287. (**B**) The residues of the catalytic triad and the oxyanion hole in the *p-*Np bound Est22 are shown as stick models. The *p-*Np molecule is shown as a stick model. (**C**) The residues of the catalytic triad and the oxyanion hole in the *p-*Np bound S188A mutant are shown as stick models. The *p-*Np molecule that shown as a stick model forms hydrogen bonds with residues G108, G109, and G189. (**D**) Structural superimposition of the *p-*Np bound wild type Est22 and S188A mutant is shown in cartoon view. The *p-*Np1 and *p-*Np2 molecules are from wild type Est22 structure, while the *p-*Np3 molecule from the mutant is shown in stick model with electronic map surface. (**E**) Structural superimposition of the four crystal structures. The apo-Est22, *p-*Np bound Est22, S188A, and *p-*Np bound S188A structures are colored with magentas, yellow, pink, and cyan respectively. (**F**) The proposed active site of Est22 binding with C4 (*p*-nitrophenol butyrate) is built by AUTODOCK program. A hydrogen-bond network between active site and substrate C4 is shown in black. All the electronic maps are contoured to 1.5 σ at 2*F*_*o*_*-F*_*c*_ map.

The substrate-binding pocket of Est22 forms a deep and narrow gorge, which is distant from the dimeric interface. It can be divided into three regions, including the bottom, the central and the entrance part. The bottom region is composed of the loop region and the catalytic triad residues Ser188, His317, and Asp287 (Fig. 4a). Several large hydrophobic regions in the middle part are formed by the side chains of residues Leu113, Tyr219, Leu240, Leu289, Ile321, and Phe322. At the entrance of the binding pocket, Arg49, Phe55 (α3 helix), and Asp241 (in the loop between α7 and α8 helices) could facilitate the entry of substrate via hydrophobic and electrostatic interactions. Five acidic residues were found in the entrance, including Glu43, Glu44, Asp50 and Asp57 on the α3 helix, and Glu187 on the bottom of binding pocket.

In the catalytic sites of Est22, Ser188 is a nucleophile residue, His317 is the proton acceptor/donor, while Asp287 helps to stabilize the His317 residue. Within the conserved penta-peptide sequence motif Gly-X-Ser-X-Gly^16^, Ser188 is located at the apex of the nucleophile elbow^1^, a sharp turn connecting β5 and α6. Ser188 is ~25 Å away from the protein surface, which could protect the active site from being exposed to water. The Ser188 conformation is stabilized by a hydrogen bond between the O^γ^ atom of Ser188 and the N^ε2^ atom of His317. Asp287 and His317 are stabilized by the hydrogen bond network between the carboxyl edge of β7 and β8.

One oxyanion hole was found in Est22, which was composed of residues Gly108, Gly109, and Gly189. Gly108 and Gly109, located in the His-Gly-Gly-Gly motif (residues 106–109), is usually conserved in HSL family (Fig. 4a). The main-chain nitrogen atoms of the oxyanion hole donate hydrogen to the cleaved substrate, which stabilizes the negative charges on the tetrahedral intermediates arising from the nucleophilic attack of Ser188 to substrate during hydrolysis^14^. The hydrogen bond network contributes to the configuration of Gly109 and Gly189. For example, Gly109 interacts with Gln111 and Ser112 via hydrogen bonds between the O atom of Gly109 and the N atoms of Gln111 and Ser112.

### Structural based mutation study of Est22

In order to investigate the relationship between key residues and the hydrolysis activity of Est22, we performed mutagenesis analysis for the catalytic triad residues Ser188, His317, and Asp287, and residue Ser170 that is located on α5 helix. The native Est22 and mutants Ser188Ala, Ser170Ala, Ser188Ala/Asp287Ala, and Ser188A/His317A were eluted as a dimer on gel-filtration profile, whereas Asp287Ala, His317Ala, Asp287Asn, His317Phe and His317Leu shifted from dimeric to polymeric form (Fig. 1c,e). As immobilized enzymes (aggregates) can be served as highly efficient and specific catalysts in industry application^9^,^17^, we compared the enzymatic activities of the mutants with wild type Est22. Esterase activity was examined using different substrates, including *p*-nitrophenyl acetate (C2), *p*-nitrophenyl butyrate (C4) and *p*-nitrophenyl hexanoate (C6) at pH 7.5 and 40 °C (Fig. 1d,f). A significant decrease in enzymatic activity was observed for mutants of residues in active center, while the activity of Ser170Ala decreased 40% on C4 but increased 10% on C6 than native Est22. These results clearly show that the three amino acids (Ser188, His317, and Asp287) are active in the center and are essential for the biological function of Est22.

As the hydrolysis process is superfast, it is difficult to get to the intermediate state by crystallization. However, we successfully obtained several types of Est22 crystal structures, including the *p-*Np bound Est22 (PDB ID: 5HC0), the *p-*Np bound mutant S188A (PDB ID: 5HC2), S188A (PDB ID: 5HC5), and S170A (PDB ID: 5HC3) (Table 1). Structure superimposition of the wild type Est22 with its mutants revealed that the structures are almost identical, as the RMSD of Cα atom values are 0.163 Å (*p-*Np bound Est22), 0.156 Å (S188A), 0.196 Å (*p-*Np bound S188A), and 0.190 Å (S170A), respectively. In the wild type Est22, His317 forms hydrogen bonds with Ser188 and Asp287, while the bond is abolished in the mutant S188A structure (Fig. 4a–d). Meanwhile, the configuration and space site of the product *p*-Np is totally different between the wild type and mutant structures. The product *p*-Np is located in the substrate-binding pocket, but does not occupy the active site, which indicates that the product transferred out of the active site after hydrolysis was done (Fig. 4b,e). In the *p*-Np bound mutant S188A structure, one *p*-Np located on the active center, which could result from the enzymatic activity abolishment (Fig. 1d). The *p*-NP in S188A mutant was stabilized by hydrogen-bonding interactions with three amino acids of oxyanion hole, Gly108, Gly109, and Gly189, whereas the *p*-NP in wild type Est22 only interacted with Ser188 via hydrogen bond (Fig. 4a–d).

To investigate the interaction between the Est22 and the substrate, the complex structure was simulated by AUTODOCK program^18^ using the configuration of wild type protein structure and substrate *p*-nitrophenyl butyrate (C4). Ten configuration models were obtained with the most probable configuration found at the lowest binding energy. In this configuration, the oxygen atoms on C4 interact with the amino acids (Gly108, Gly109, Gly189, Ser188, and His317) of the active center and oxyanion hole via hydrogen and covalent bonds (Fig. 4f). When the four structures were superimposed together (Fig. 4e), it clearly showed that the product *p*-Np orientation changed between the wild type and mutants Est22. Combined with the conformations in *p*-Np bound wild type Est22 or mutant S188A, it was proposed that His317 could be protonated by Ser188, thus, remaining Ser188 could act as a nucleophile to attack the carbonyl carbon atoms of the ester, forming a tetrahedral shape of intermediates. The intermediate was stabilized by an oxygen hole, which in turn leads to the carbonyl of the tetrahedral intermediate to transfer the proton to N atom of His317. Finally, the carboxylate moiety leaves the enzyme when the hydrolysis is finished^14^.

### The dimeric interface of Est22

**Figure 5:**
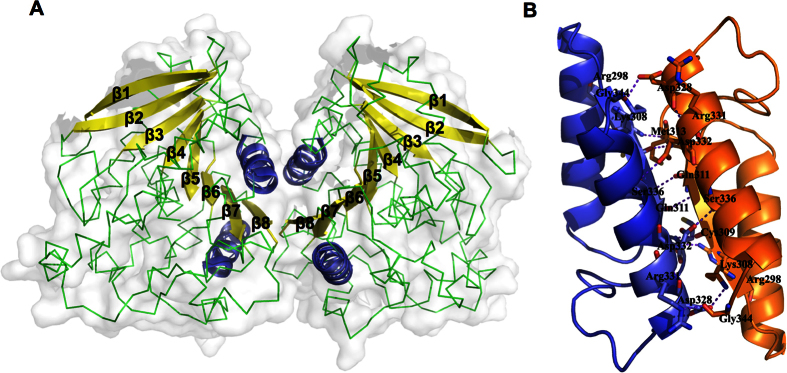
Representation of the dimeric interface in Est22. (**A**) The molecular surface of the dimeric Est22 is colored with light gray. The two interacting helices are colored with dark blue. The continuous β sheets are depicted in cartoon with bright yellow, while other parts are shown in green. (**B**) A hydrogen-bond network between two α helices of Est22 subunit is shown in a stick model colored with dark blue and bright orange. The hydrogen bonds are depicted as dashed purple lines.

**Figure 6:**
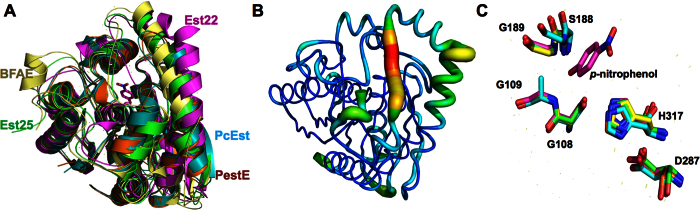
A structural comparison of Est22 with other homologous esterases. (**A**) The structural superposition of Est22 (magenta, PDB ID: 5HC0), EST25 (green, PDB ID: 4J7A; RMSD of 1.7 Å on 323 Cα atoms), BFAE (pale yellow, PDB ID: 1JKM; RMSD of 2.2 Å on 326 Cα atoms), PcEst (teal, PDB ID: 3ZWQ; RMSD of 2.2 Å on 295 Cα atoms), and PestE (orange, PDB ID: 2YH2; RMSD of 2.1 Å on 294 Cα atoms). (**B**) The B-factor distribution of Est22. The wider and redder tubing indicates higher B-factor. (**C**) The comparison of detailed binding and catalytic active sites among the five homologous proteins. The product *p*-Np and five residues are depicted as a stick model. Colors are labeled as the same with (**A**).

The wild type Est22 has four molecules in the asymmetric unit. The four monomers in an asymmetric unit were arranged as a dimer of dimers (Fig. 5a). Each dimer in the asymmetric unit was related by two-fold noncrystallographic symmetry, which shared similar horseshoe-shaped cap domain with previous reported Est25, whereas differing from other esterases in its topological confirmation (Figs 5a and 6a). The monomers in the asymmetric unit were almost identical, as the pairwise root-mean-square deviation (r.m.s.d.) values among all Cα atoms of the four molecules in the asymmetric unit ranged from 0.153 Å to 0.165 Å. The Est22 dimer was formed by edge-to-edge interactions via two core β-sheets of each monomer with two-fold symmetry. Its interface was formed largely by α10, α11 helices, and β8 stand, which included 2 salt bridges and 11 hydrogen bonds. One salt bridge was between residues Lys308 (at β8 sheet) and Asp328 (at α11 helix), while the other was between Glu285 (at the loop between β7 and α10) and Arg298 (at α10 helix). 11 hydrogen bonds located between the residues of Glu285, Arg298, Lys308, Cys309, Gln311, Met313, Arg331, Asp332, Asp328, Ser336, and Gly344 (Fig. 5b). The residues Phe280, Ile294, Tyr297, Arg310, Gly312, and Val329 appeared in non-bonded contacts at the dimeric interface. To achieve catalytic activity and maintain thermostability, dimerization of two monomers is essential in HSL family enzymes^19^,^20^,^21^. In addition, we mutated 11 amino acid residues that are involved in the dimeric surface (E285A, R298A, K308A, C309A, Q311A, M313A, D328A, R331A, D332A, and S336A) (Fig. 1e,f). The gel filtration profiles and enzymatic activities of these mutants clearly explain the reason why Est22 has hyperthermostability and very high enzymatic activity. Notably, disruption of dimerization has successfully been employed as a strategy for the development of effective inhibitors^22^,^23^.

### Structural comparison of Est22 with other esterases

**Figure 7:**
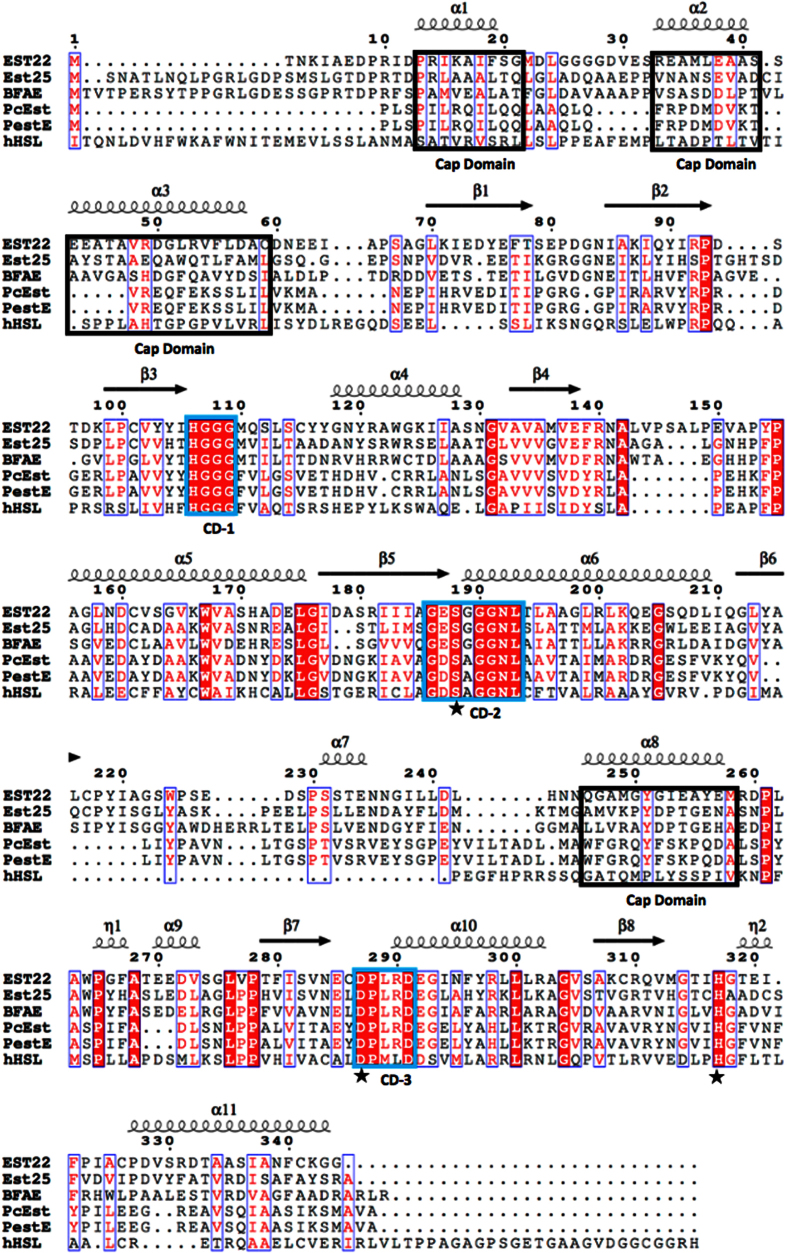
Multiple sequence alignment of Est22 and four structurally homologous enzymes, EST25 (PDB code 4J7A from environmental samples), BFAE (PDB code 1JKM from *B. subtilis*), PcEst (PDB code 3ZWQ from *P. calidifontis*), PestE (PDB code 2YH2 from *P. calidifontis*), and human HSL (hHSL). Four helices (α1, α2, α3, and α8) are shown in black boxes labeled with cap domain at the bottom. Identical and highly conserved residues are colored in red and white, respectively. Enzyme activity site triad S188, D287 and H317 are labeled with asterisk. Three highly conserved motifs are depicted by light blue box and labeled with CD-1, CD-2 and CD-3 at the bottom.

The structural similarity analysis was carried out using DALI server search, which showed that Est22 has significant structural homologies to previously reported esterases, including Est25 from metagenomic library (PDB code 4J7A), BFAE from *B. subtilis* (PDB code: 1JKM), PcEst from *P. calidifontis* (PDB code 3ZWQ), and PestE from *P. calidifontis* (PDB code 2YH2)^10^,^16^,^24^. However, the primary sequence between Est22 and these esterases are low, which ranges from 27% to 39%, the RMSD values of Cα atom are 1.7 Å (for 323 residues in Est25), 2.2 Å (for 326 residues in BFAE), 2.2 Å (for 295 residues in PcEst), and 2.1 Å (for 294 residues in PestE), respectively. This clearly shows that Est22 shares high similarity with other members of α/β-hydrolase fold family. Superimposing Est22 on the structures of Est25, BFAE, PcEst, and PestE revealed similar features of overall folds between these HSL family esterases (Fig. 6a,b). The core residues of the α/β-hydrolase including the catalytic triad are highly conserved (Figs 6c and 7). Several conserved sequence motifs, such as ^106^HGGG^109^, ^186^GXSXG^190^ and ^287^DPXXD^291^, widely exist in the catalytic center of HSL family members, suggesting Est22 shares the same catalytic mechanism with that of HSL family members. The structural differences are largely in the three loop regions, which are located between α1 and α2, β4 and α5, β 6 and α7, respectively (Fig. 7). Several reported mutants (P146Q and a 19bp frameshift deletion in exon 9) are located on the outside of the alpha/beta hydrolase domain of HSL, which could function via regulating the enzymatic activity of HSL^8^,^25^.

## Conclusions

Here we report the structural and functional analysis of an HSL family esterase Est22 from the deep sea. The analysis of the biochemical properties and the structural information of Est22 and its mutants greatly improve our understanding of the catalytic mechanism of the α/β-hydrolase fold family. As Est22 shares conserved active sites and essential motifs for hydrolysis of HSL family, our study could provide a novel way for developing new HSL inhibitors and broadening the applications as a biocatalyst in environment and industry.

## Materials and Methods

### Protein expression and purification

cDNA fragment encoding full length Est22 (1–344 aa) was amplified by PCR method from a deep-sea metagenomic library^15^. The cDNA of Est22 was cloned into pET28b vector with a N-terminal 6xHis tag. The wild type and mutants of Est22 plasmids were transformed into *E. coli* Rosetta (DE3) cells for protein expression. *E. coli* cells were cultured in LB medium with 50 ug/ml kanamycin and 34 ug/ml chloramphenicol at 37 °C. Isopropyl 1-thio-β-D-galactopyranoside (IPTG, 0.5 mM) was added into cells to induce protein expression at 25 °C for 8 h when the OD_600_ reached 0.6–0.8. Then the cells were harvested by centrifugation at 6000 rpm for 10 min at 4 °C. The cell pellets were resuspended in a lysis buffer (50 mM Tris-HCl, 500 mM NaCl, 10 mM imidazole, 5% Glycerol, 2 mM βME, pH 8.0) and disrupted using a high-pressure homogenizer (JNBIO, China). The cell debris was removed by centrifugation at 17000 rpm for 60 min at 4 °C. The supernatant was purified using a His Trap^TM^ HP column (GE Healthcare) and was eluted in a buffer (50 mM Tris-HCl, 500 mM NaCl, 250 mM imidazole, 5% Glycerol, pH 8.0). The protein was further purified by gel filtration using a Superdex 200 16/600 column (GE Healthcare) in a gel filtration buffer (20 mM Tris-HCl, 100 mM NaCl, 2 mM DTT, pH 7.4)^26^. The purity of the Est22 protein was confirmed by SDS-PAGE and concentration was determined by the method of Bradford with bovine serum albumin (BSA) as a standard.

### Multi-angle light scattering (MALS)

The oligomeric state of Est22 was identified by multi-angle light scattering method (WH2-06, Wyatt) in National Center for Protein Science Shanghai (NCPSS). In brief, 20 ul of 1 mg ml^−1^ Est22 was loaded into the wtc030s5 column (Wyatt) with the elution buffer (20 mM Tris-HCl, 100 mM NaCl, pH 7.4). The collected data was processed with Astra 6 software (Wyatt).

### Mutagenesis

Mutants S170A, S188A, D287A, H317A, D287A/S188A, S188A/H317A, A287N, H317F, H317L, E285A, R298A, K308A, C309A, Q311A, M313A, D328A, R331A, D332A, and S336A were generated by whole-plasmid PCR in 18-cycle reaction with steps at 98 °C for 10 s, 55 °C for 30 s, and 72 °C for 3 minutes per cycle. After digestion with enzyme DpnI, the PCR products were transformed into *E. coli* Top10 cells. The positive constructs were determined by DNA sequencing.

### Biochemical characterization of Est22

The standard reaction was carried out with the appropriate amount of purified Est22 in 1 ml buffer containing 100 mM Tris-HCl (pH7.5) and 1 mM *p*-nitrophenyl acetate^27^. The activity of the enzyme was determined by measuring the amount of released *p*-nitrophenol at 40 °C at 405 nm using DU800 UV/Visible spectrophotometer (Beckman, USA). All samples were measured in triplicate and corrected for autohydrolysis of the substrate.

The molar extinction coefficient of *p*-nitrophenyl is 15,000 M^−1 ^cm^−1^ at 405 nm. The kinetic parameters were determined by using *p*-nitrophenyl acetate as substrate at different concentrations ranging from 0.05 to 4 mM. The values of *Km* and *Vmax* were analyzed by the Michaelis-Menten equation using GraphPad Software (GraphPad Inc., USA).

The substrate specificity was determined by *p*-nitrophenyl esters with various chain lengths, including *p*-nitrophenyl acetate (C2), *p*-nitrophenyl butyrate (C4), *p*-nitrophenyl hexanoate (C6), *p*-nitrophenyl octanoate (C8), *p*-nitrophenyl decanoate (C10), *p*-nitro-phenyl laurate (C12), *p*-nitrophenyl myristate (C14), and *p*-nitrophenyl palmitate (C16). Except C6 ester (TCI, Japan), all the other esters were purchased from Sigma. The optimum temperature of the enzyme activity was determined ranging from 20 to 55 °C. Thermostability of the enzyme was analyzed by measuring the residual activity at the optimum temperature after incubating the enzyme for 30 minutes at 10, 20, 30, 40, 50, and 60 °C in the absence of the substrate^15^.

A universal buffer was used for the determination of optimum pH. The buffers included 100 mM citrate buffer (pH 3.0–6.5), 100 mM potassium phosphate buffer (pH 6.5–7.5), 100 mM Tris-HCl buffer (pH 7.5–9.0) and 50 mM CHES buffer (pH 9.0–10.0). The influences of cations on enzyme activity were examined in the presence of 10 mM Ba^2+^, Ca^2+^, Co^2+^, Cu^2+^, Mg^2+^, Mn^2+^, Ni^2+^, Sr^2+^, Zn^2+^, and the chelating agent EDTA. The effect of organic solvents was examined by using 15% acetone, acetonitrile, alcohol, dimethylformamide (DMF), dimethyl sulfoxide (DMSO), isopropanol or methanol respectively. The effect of the detergents was determined by using 1% Tween 20, Tween 80, Triton X-100, or SDS. All measurements were performed in 100 mM Tris-HCl buffer (pH 7.5), and the activity of the enzyme, without additives, was defined as 100%.

### Crystallization and data collection

Est22 was crystallized by hanging- and sitting-drop vapour-diffusion methods by mixing 1 μl of 20 mg ml^−1^ protein with 1 μl reservoir solution at 4 °C and 20 °C respectively. The diffraction quality crystals of Est22 were grown in a reservoir solution containing 0.1 M HEPES sodium (pH 7.5) and 1.5 M Lithium sulfate monohydrate. The pNp bound Est22 crystals were grown in the condition of 0.1 M MES/sodium hydroxide (pH 5.5), 0.2 M calcium acetate and 15% PEG8000. The S188A mutant with or without pNp-bound crystals were both grown in the condition of 1 M sodium citrate (pH 8.0), 0.1 M imidazole. The S170A crystals were grown in the condition of 0.2 M sodium tartrate dehydrate (pH 7.3), 20% PEG3350. All of the crystals were briefly soaked in a cryoprotectant solution consisting of 25% (v/v) glycerol dissolved in their corresponding mother liquors before being flash-cooled directly in a liquid-nitrogen stream at 100 K. The X-ray diffraction data were collected at the BL17U and BL19U beamlines of Shanghai Synchrotron Radiation Facility (Shanghai, China). Intensity data were integrated and scaled using HKL2000^28^ or HKL3000.

### Structure determination and refinement

The crystal structure of wild type Est22 was determined by molecular replacement using the crystal structure of Est25 (PDB code 4J7A)^10^ as the search model. The other structures were determined by molecular replacement using Est22 structure as the search model. Cycles of refinement and model building were carried out by using REFMAC^29^ and COOT^30^ programs until the crystallography R-factor and free R-factory values reached to satisfied range. The quality of the final model was evaluated with PROCHECK^31^. All of the structures were displayed and analyzed using PyMOL program^32^. The simulation of Est22 with its substrate was carried out with AUTODOCK program^18^. The collected data and refinement statistics are summarized in Table 1.

## Additional Information

**Accession codes**: The coordinates and structural factors had been deposited in the Protein Data Bank with accession codes 5HC4 (Est22), 5HC0 (Est22 bound with p-Np), 5HC2 (S188A bound with p-Np), 5HC3 (S170A), and 5HC5 (S188A) respectively.

**How to cite this article**: Huang, J. *et al*. Structural insights of a hormone sensitive lipase homologue Est22. *Sci. Rep.* **6**, 28550; doi: 10.1038/srep28550 (2016).

## Figures and Tables

**Table 1:** Statistics for data collection and refinement.

**Data collection**	**Est22**	**Est22+** ***p*****-Np**	**Est22** (**S188A**)	**Est22** (**S188A**)** + *****p*****-Np**	**Est22** (**S170A**)
Wavelength (Å)	0.9785	0.9785	0.9778	0.9778	0.9778
Resolution range (Å)	50.0-2.00 (2.03-2.00)^a^	50.0-1.40 (1.45-1.40)	50.0-2.43 (2.47-2.43)	50.0-1.98 (2.01-1.98)	50.0-2.40 (2.44-2.40)
Space group	P 2_1_ 2_1_ 2_1_	C2	P 2_1_ 2_1_ 2_1_	P 2_1_ 2_1_ 2_1_	P 2_1_ 2_1_ 2_1_
Cell dimensions (Å,°)	a = 81.24, b = 121.81,c = 152.52, α = β = γ = 90	a = 121.82, b = 57.22,c = 54.58, α = γ = 90,β = 97.46	a = 81.47, b = 121.80,c = 150.00, α = β = γ = 90	a = 81.65, b = 121.93,c = 150.29, α = β = γ = 90	a = 81.16, b = 121.68,c = 150.49, α = β = γ = 90
Unique reflections	95761	66154	49689	103584	58524
Completeness (%)	98.7 (98.8)	96.2 (93.9)	96.2 (86.6)	100 (100)	100 (100)
R_merge_ (%)^b^	6.4 (77.7)	10.6 (52.3)	17.2 (35.4)	14.5 (83.1)	10.9 (57.4)
I/σ (I)	26.19 (2.27)	16.80 (4.24)	8.66 (2.66)	25.76 (3.24)	17.4 (3.6)
Wilson *B* -factor	26.67	12.99	34.15	26.51	33.61
Refinement statistics					
Resolution range (Å)	39.22-2.00 (2.07-2.00)^a^	38.47-1.40 (1.45-1.40)	47.28-2.43 (2.47-2.43)	50.0-1.98 (2.01-1.98)	37.62-2.40 (2.49-2.40)
R_work_^c^/R_free_ (%)^d^	17.20/22.96	14.27/16.68	17.79/22.80	15.31/18.39	15.29/20.18
No. atoms	11334	3122	10550	11528	10837
No. residues	1364	338	1357	1371	1359
No. *p-*Np	0	2	0	4	0
No. glycerol	1	4	0	1	4
No. water molecules	1141	508	199	1020	474
RMSD					
Bond lengths (Å)	0.019	0.038	0.012	0.007	0.014
Bond angles (°)	1.90	2.38	1.56	0.85	1.63
Average B-factor (Å^2^)	33.20	17.44	39.67	28.72	37.68
Ramachardran favored (%) afavored	95.3	96.8	95.1	95.7	95.6
Ramachardran outliers (%)	0.3	0	0.2	0.2	0.1
Rotamer outliers (%)	0.7	0.7	0.6	0.6	0.5
Clash core	4.68	5.20	1.68	2.18	2.70
MolProbity score	1.57	1.48	1.44	1.29	1.37
PDB code	5HC4	5HC0	5HC5	5HC2	5HC3

^a^Values in parentheses are for the highestresolution shell.

^b^R_merge_ = ∑|I_i_ − <I> |/∑|I|, where Ii is the intensity of an individual reflection and is the average intensity of that reflection.

^c^R_work_ = ∑||F_o_| − |F_c_||/∑|F_o_|, where F_o_ and F_c_ are the observed and calculated structure factors for reflections, respectively.

^d^R_free_ was calculated as R_work_ using the 5% of reflections that were selected randomly and omitted from refinement.
